# Cathodically Pretreated AuNPs–BDD Electrode for Detection of Hexavalent Chromium

**DOI:** 10.3390/mi11121095

**Published:** 2020-12-11

**Authors:** Yuhao Xu, Chenyu Xiong, Chengyao Gao, Yang Li, Chao Bian, Shanhong Xia

**Affiliations:** 1 State Key Laboratory of Transducer Technology, Aerospace Information Research Institute, Chinese Academy of Sciences, Beijing 100190, China; xuyuhao15@mails.ucas.ac.cn (Y.X.); xiongchenyu16@mails.ucas.ac.cn (C.X.); gaochengyao.ok@gmail.com (C.G.); yangli@mail.ie.ac.cn (Y.L.); 2 School of Electronic, Electrical and Communication Engineering, University of Chinese Academy of Sciences, Beijing 100049, China

**Keywords:** boron-doped diamond electrode, gold nanoparticles, cathodic stripping voltammetry, chromium (VI), electrochemical sensor

## Abstract

Hexavalent chromium (Cr (VI)) has strong oxidizing properties and can result in strong carcinogenic effects on human bodies. Therefore, it is necessary to detect hexavalent chromium sensitively and accurately. This article proposes the gold nanoparticles (AuNPs)–boron-doped diamond (BDD) electrode for the direct determination of chromium with a green and simple detection process by cathodic stripping voltammetry. Gold nanoparticles are used to enhance the detection performance toward Cr (VI). The effect of different pretreatment methods on electrode modification has been studied, and the detection parameters have been optimized. With the optimized conditions, the AuNPs–BDD electrode presents a good linear behavior in a Cr (VI) concentration range of 10 to 1000 μg/L. A low limit of detection of 1.19 μg/L is achieved. The detection process is simple and environmentally friendly. The sensor has been tested for the detection of Cr (VI) in a real water sample with satisfactory results, which indicates potential application of the AuNPs–BDD electrode for the sensitive and onsite detection of Cr (VI).

## 1. Introduction

Due to the strong biological toxicity and accumulation of heavy metal, heavy metal pollution can cause serious threats to human health. Hexavalent chromium (Cr (VI)) can oxidize the biological molecules in contact with it, and it has strong stimulating and corrosive effects on the skin and mucous membranes of human body. It can cross-link with the DNA in the nucleus, which leads to rearranging of DNA strands. So, Cr (VI) can cause great harm to the human body [1,2]. Due to the rapid development of industry and imperfect environmental protection technology, a large amount of Cr (VI) is discharged into surface water. Therefore, it is very important to establish accurate and sensitive monitoring of hexavalent chromium in water.

At present, conventional detection methods for Cr (VI) mostly need large-size detection equipment, such as atomic absorption spectrometry [3,4,5], inductively coupled plasma mass spectrometry [6,7,8], the X-ray fluorescence method [9,10,11,12], and so on. These methods are complicated and costly. Compared with traditional detection methods, the electrochemical method has the advantages of simple operation, low detection cost, and high detection sensitivity, which make it suitable for online analysis and the monitoring of Cr (VI) in water [12]. At the same time, the electrochemical method is suitable for the speciation analysis of chromium, and it can distinguish between Cr (VI) and Cr (III) without additional separation steps [13].

At present, many electrochemical detection methods for Cr (VI) require complexing agents or acidification steps in the detection process. The use of bismuth-based electrodes for Cr (VI) detection can achieve a low limit of detection, which requires the addition of complexing agents (such as diethylenetriamine pentaacetic acid) and catalysts (such as nitrate) [14,15,16]. Gold-based electrodes could be used to detect Cr (VI), and it needs to be performed in an acidic solution to achieve maximum current response [17,18,19], which complicates the detection procedure.

Gold nanoparticles have been widely applied in fuel cells, optical, and electrochemical fields [20,21,22]. As one of the excellent nanomaterials, gold nanoparticles have a large specific surface area, excellent catalytic performance, and good conductivity, which can improve the performance of electrochemical Cr (VI) detection [23,24]. In recent years, the carbon materials have attracted attention for the analysis of Cr (VI). Taking into account that boron-doped diamond electrode (BDD) has showed excellent electrochemical properties [25,26,27] such as low background current, wide potential window, high stability, and non-toxicity, which can improve performance of electrochemical detection and facilitate the realization of electrode surface renewal, the BDD electrode may be an excellent option in the trace detection of Cr (VI) [28,29,30,31]. Combining gold nanoparticles with boron-doped diamond electrode for Cr (VI) analysis not only takes advantage of the high catalytic properties and large active surface of gold nanoparticles but also makes use of low background current and high electrochemical stability of the BDD electrode to reduce the limit of detection and improve the sensitivity detection and stability of the sensor.

Therefore, this paper presents the gold nanoparticles (AuNPs)–boron-doped diamond (BDD) electrode to achieve the sensitive and rapid detection of Cr (VI) and studies the influence of cathodic and anodic pretreatment for a BDD electrode on the modification of the electrode. The detection parameters such as pH have been optimized. The detection process is environmentally friendly. According to the results, the AuNPs–BDD electrode can provide a possibility for the onsite and sensitive detection of Cr (VI).

## 2. Materials and Methods

### 2.1. Instruments, Reagents, and Materials

The electrochemical experiments were performed by using a Reference 600 Electrochemical Workstation (Gamry Instruments, Warminster, PA, USA) with a three-electrode system. The platinum electrode and the Ag/AgCl electrode were the counter electrode and reference electrode individually. The scanning electron microscope (SEM) images were obtained by using an S-4800 Field Emission Scanning Electron Microscope (Hitachi, Japan). Deionized water was obtained from a Millipore Direct-Q 3 UV system (Merck Millipore, Billerica, MA, USA). All reagents were of analytical grade. Hexavalent chromium ion standard solution was purchased from Macklin Inc. (Shanghai, China). Acetone, ethanol, and sulfuric acid were obtained from Beijing Chemical Works (Beijing, China). HAuCl_4_ and sodium acetate were purchased from Sinopharm Chemical Reagent Co., Ltd. (Shanghai, China).

### 2.2. Fabrication of BDD Electrode

According to the previous research of our group, the BDD electrode was prepared by the hot filament chemical vapor deposition method [27]. The silicon substrate is heavily doped, which ensures a good ohmic contact between the boron-doped diamond film and silicon substrate. Before hot filament chemical vapor deposition, the polishing and ultrasonic cleaning of silicon substrate was performed. The deposition was carried out with a hot filament temperature of 2000 °C, a deposition pressure of 2.5 kPa, a hydrogen flow rate of 200 mL/min, and an acetone flow rate of 60 mL/min. The deposition time was 5 h. The thickness of the BDD film is 3–5 μm, approximately. The area of the BDD electrode is 12.56 mm^2^.

### 2.3. Modification of BDD Electrode

Before modification of the BDD electrode, the anodic pretreatment was achieved by applying a potential of +3 V to the BDD electrode for 120 s in a 0.5 mol/L H_2_SO_4_ solution to a clean electrode surface and make surface termination become oxygen termination. Then, the cathodic pretreatment was achieved by applying a potential of −3 V to the BDD electrode for 300 s in a 0.5 mol/L H_2_SO_4_ solution to make suface termination become hydrogen termination. The electrochemical deposition of gold nanoparticles was carried out in 0.5 mol/L H_2_SO_4_ containing 2 mmol/L HAuCl_4_. By the constant potential method, the gold nanoparticles were electrodeposited effectively on the surface of the BDD electrode with a potential of −0.2 V for a certain time. Subsequently, cyclic voltammetry (CV) was performed from 0 to 1.5 V with a scan rate of 50 mV/s in 0.5 mol/L H_2_SO_4_ until a stable voltammetry curve was obtained, the cleaning and activation of an electrode was achieved. After rinsing with deionized water, the modification process of the AuNPs–BDD electrode was completed.

### 2.4. Detection and Analysis of Cr (VI)

Cr (VI) analysis and detection were performed by cyclic voltammetry and cathodic stripping voltammetry. The cyclic voltammetry was performed with 50 mV/s for 5 cycles in 0.1 mol/L sodium acetate buffer. After preconcentration with a certain preconcentration potential and time, the Cr (VI) was detected by square wave voltammetry with a potential of 0.6 to 0 V (pulse size = 25 mV, frequency = 25 Hz, step size = 5 mV). After each measurement, a potential of 0 V was applied to the working electrode for 30 s to eliminate the residual Cr (VI) on the electrode surface.

## 3. Results and Discussion

### 3.1. AuNPs–BDD Electrode Detection Principle

**Figure 1:**
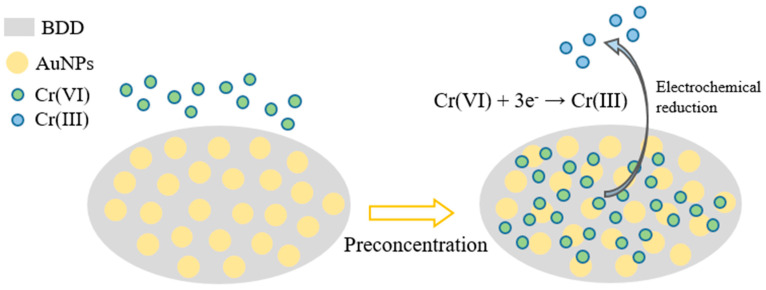
Working principle of Cr (VI) detection by gold nanoparticles (AuNPs)–boron-doped diamond (BDD) electrode.

**Figure 2:**
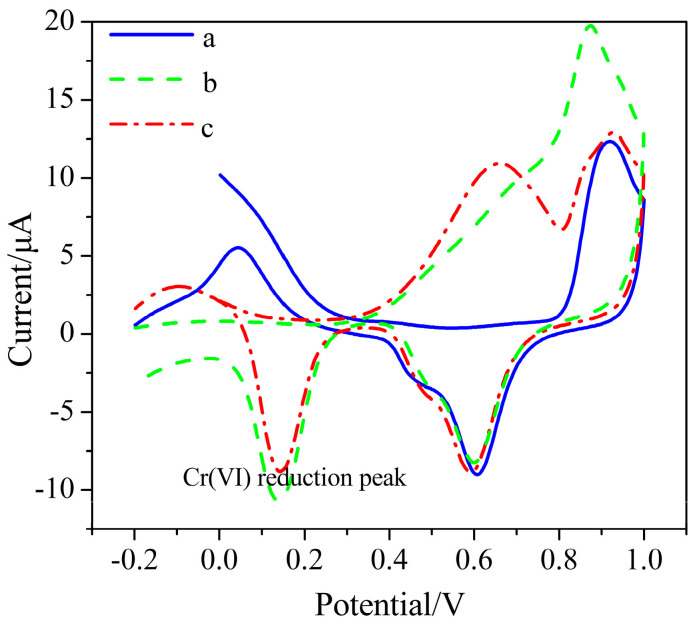
The cyclic voltammetry of the AuNPs–BDD electrode in absence of (a) and in presence of 100 μmol/L hexavalent chromium (Cr (VI)) in the 1st cycle (b) and 5th cycle (c).

This paper realizes the direct detection of Cr (VI) utilizing the electrochemical reduction of Cr (VI) on the AuNPs–BDD sensitive electrode, which is environmentally friendly and has a simple detection process. The principle of Cr (VI) detection using an AuNPs–BDD electrode is shown in Figure 1. The negatively charged Cr (VI) in the solution is adsorbed on the electrode surface by electrostatic adsorption when the electrode is applied with a positive potential. The oxidizing Cr (VI) on the electrode surface is electrochemically reduced to Cr (III), as described in Figure 1. The direct detection of Cr (VI) is achieved by measurement of the reduction current. As shown in Figure 2, the CV curve of the AuNPs–BDD electrode in Cr (VI) solution shows a significant Cr (VI) reduction peak current, indicating the three-electron reduction of Cr (VI) on the surface of the AuNPs–BDD electrode. Compared with the weak oxidation peak in the 1st cyclic voltammetry curve, the oxidation peak appears in the 5th cyclic voltammetry curve around +0.6 V, which may be due to the re-oxidation of the reduced chromium during the cyclic voltammetry.

### 3.2. AuNPs–BDD Electrode Surface Characterization

**Figure 3:**
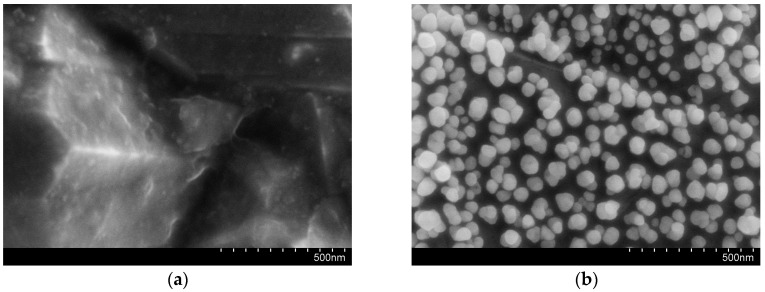
The SEM images of the BDD electrode before (**a**) and after (**b**) AuNPs deposition.

**Figure 4:**
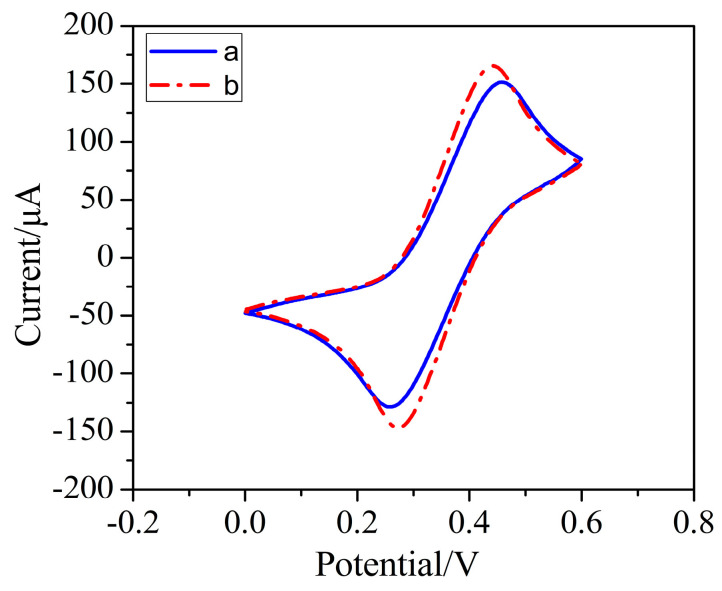
The CV curve of the BDD electrode in potassium ferricyanide before (a) and after (b) AuNPs deposition with a scan rate of 50 mV/s.

**Figure 5:**
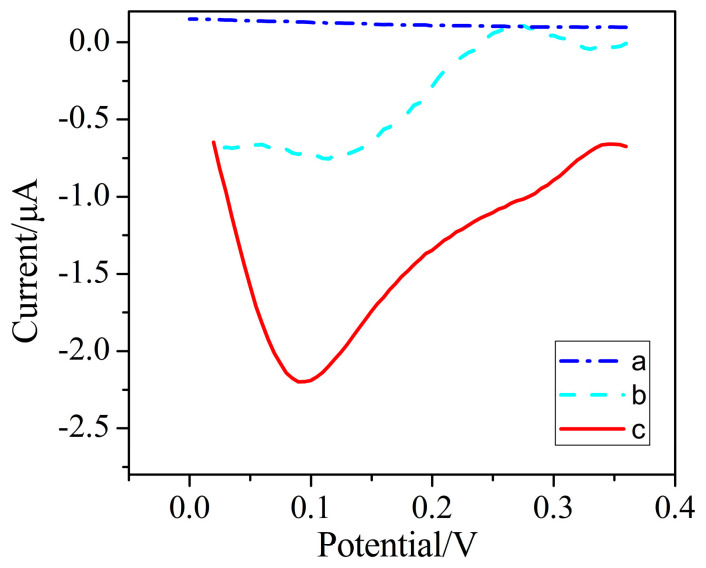
The detection current for 500 ug/L Cr (VI) of the bare BDD electrode (a), the AuNPs–Au electrode (b), and the AuNPs–BDD electrode (c).

Gold nanoparticles were deposited on the electrode surface by the electrochemical method. The SEM images of the bare BDD electrode and AuNPs–BDD electrode are shown in Figure 3. Compared with the bare BDD electrode, spherical gold nanoparticles are evenly distributed on the surface of the BDD electrode. As shown in Figure 4, the increase in redox current and decrease in redox peak potential difference is observed after the deposition of gold nanoparticles. Compared to the BDD electrode, the electrochemical activity of the AuNPs–BDD electrode for the redox of Fe(CN)_6_^3−/4−^ is enhanced by a catalytic effect and increase in active area of AuNPs on the BDD electrode surface. The results show that the gold nanoparticles have been successfully deposited on the BDD electrode. Figure 5 shows the comparison of the electrochemical performance of the bare BDD electrode, the AuNPs–Au electrode, and the AuNPs–BDD electrode for detecting Cr (VI) with the concentration of 500 μg/L. Compared with the bare BDD electrode and the AuNPs–Au electrode, an obvious increase in the reduction current intensity at the AuNPs–BDD electrode is observed, suggesting the good performance of the AuNPs–BDD electrode toward Cr (VI) detection.

**Figure 6:**
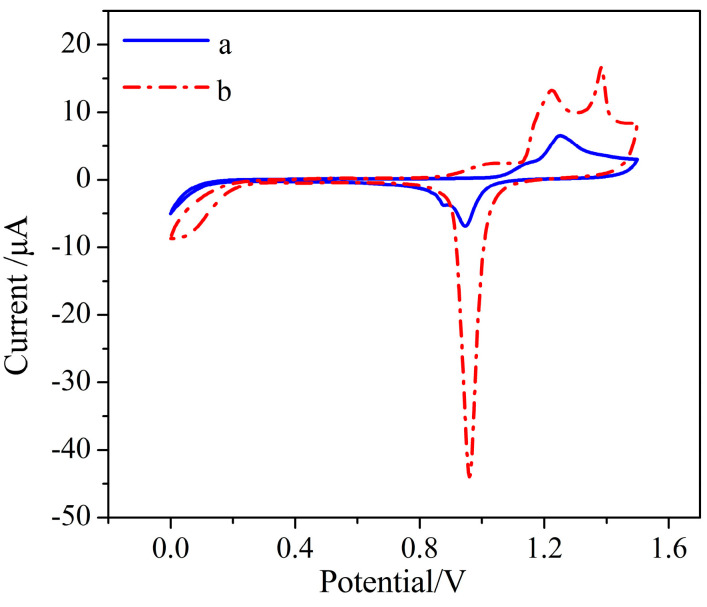
The cyclic voltammetry of the AuNPs–BDD electrode with anodic (a) and cathodic (b) pretreatment after AuNPs deposition in 0.5 mol/L H_2_SO_4_.

**Figure 7:**
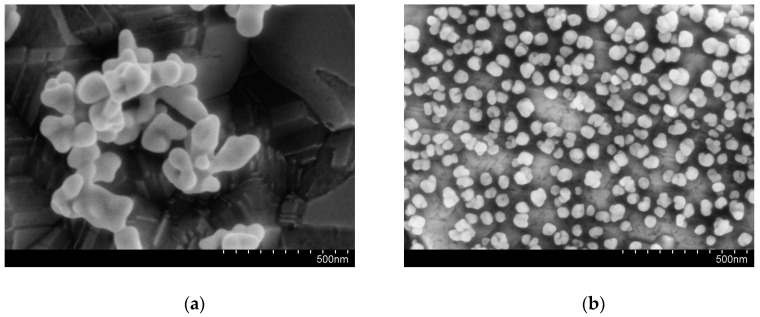
The morphology of the AuNPs–BDD electrode with anodic (**a**) and cathodic (**b**) pretreatment after deposition.

The electrochemical properties of the BDD electrode are dependent on whether the electrode surface is oxygen termination or hydrogen termination. The deposition of gold nanoparticles on the BDD electrode could be influenced by two types of surface termination. The hydrogen termination of the BDD electrode can be achieved by cathodic pretreatment, and the oxygen termination of the BDD electrode can be achieved by anodic pretreatment before the deposition of the gold nanoparticles [32,33,34]. The gold nanoparticles were deposited on the surface of these two electrodes. The AuNPs deposition after cathodic and anodic pretreatment of the BDD electrode respectively were electrochemically characterized in H_2_SO_4_ solution. The gold reduction characteristic peak in H_2_SO_4_ solution can be used to characterize the specific surface area of the electrode [35]. As shown in Figure 6, it can be seen that the gold reduction characteristic peak of the electrode after cathodic pretreatment is obviously larger than the characteristic peak of the electrode after the anodic pretreatment, indicating that the deposition of AuNPs after the cathodic pretreatment has a larger specific surface area. The morphology of the electrode surface is shown in Figure 7; the gold nanoparticles deposited after cathodic pretreatment uniformly distribute on the electrode and the gold nanoparticles deposited after anodic pretreatment sparsely distribute on the electrode surface in the form of gold nanoclusters, which shows that the electrode surface after the cathodic pretreatment is more suitable for the electrochemical deposition of AuNPs. Therefore, cathodic pretreatment for the BDD electrode is used in subsequent experiments.

### 3.3. Parameter Optimization

The detection performance of the proposed electrode is affected by detection parameters. Therefore, this article optimizes detection parameters, including the type of supporting electrolyte, pH, and AuNPs deposition time and so on. The optimized parameters are determined by comparing the reduction current of Cr (VI), and each experiment is repeated at least three times.

Several electrolytes including hydrochloric acid, nitric acid, phosphate buffer, and sodium acetate buffer were used to study the influence of supporting electrolytes. The diffusion of Cr (VI) on the electrode surface in supporting electrolytes plays an important role in the Cr (VI) detection process. Therefore, the type of supporting electrolyte has a great effect on Cr (VI) detection performance. Table 1 shows the reduction current of Cr (VI) in different electrolytes. Compared to other electrolyte solutions, the reduction current of 100 μmol/L Cr (VI) in sodium acetate buffer is large. This may be due to the facility of diffusion of Cr (VI) in sodium acetate buffer on the AuNPs–BDD electrode and the influence of electrolyte pH [17]. The reduction peak does not appear in the hydrochloric acid, which may be due to the formation of chromate complexes such as CrO_3_Cl^−^ [36,37]. Therefore, the sodium acetate buffer was selected as the supporting electrolyte solution for the detection of Cr (VI) in the subsequent experiments.

**Figure 8:**
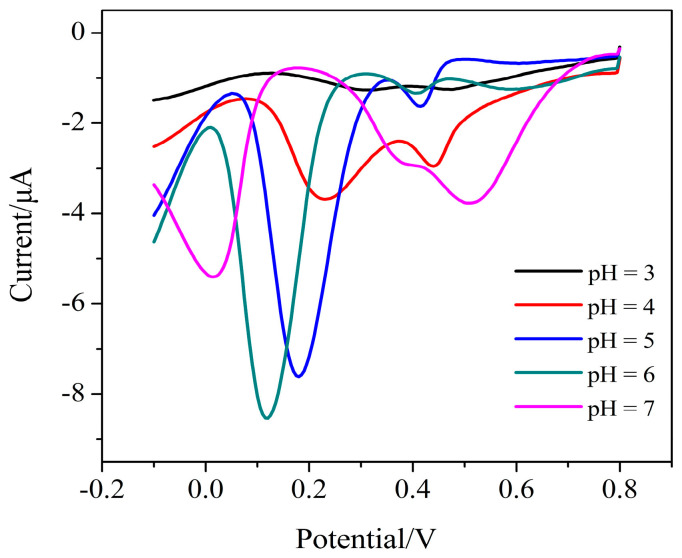
The effect of pH on the current response of Cr (VI) detection (pulse size = 25 mV, frequency = 25 Hz, step size = 5 mV, 0.1 mol/L sodium acetate buffer).

Figure 8 shows the influence of the pH on the reduction peak current in the presence of 100 μmol/L Cr (VI). The pH of the supporting electrolyte can greatly affect the shape of the reduction current curve and the value of the reduction current of Cr (VI) on the AuNPs–BDD electrode. In the pH range of 3.0–6.0, the peak current increases with the increase of pH. This may be due tothe amount of HCrO_4_^−^ gradually increases, which is the main form of Cr (VI) and has strong electrochemical activity. The maximum reduction current value at pH = 6.0 means that the amount of HCrO_4_^−^ reaches the maximum. In the pH range of 6.0–7.0, the peak current decreases as the pH value increases. Since the electrochemical activity of Cr (VI) decreases in this pH range, the sodium acetate buffer with pH = 6.0 is selected for subsequent experiments. The solution at pH = 6 tends to be neutral, which makes the detection process simple, fast, and environmentally friendly.

**Figure 9:**
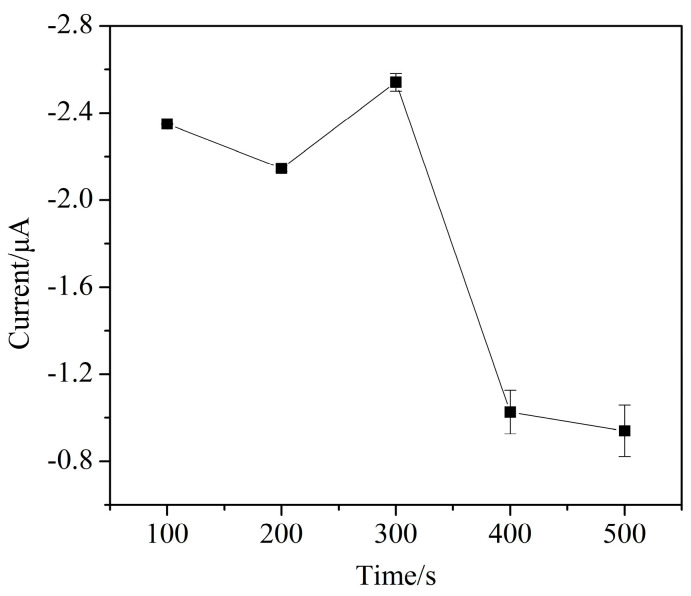
The effect of AuNPs deposition time on the current response of Cr (VI) detection (pulse size = 25 mV, frequency = 25 Hz, step size = 5 mV, 0.1 mol/L sodium acetate buffer, pH 6).

**Figure 10:**
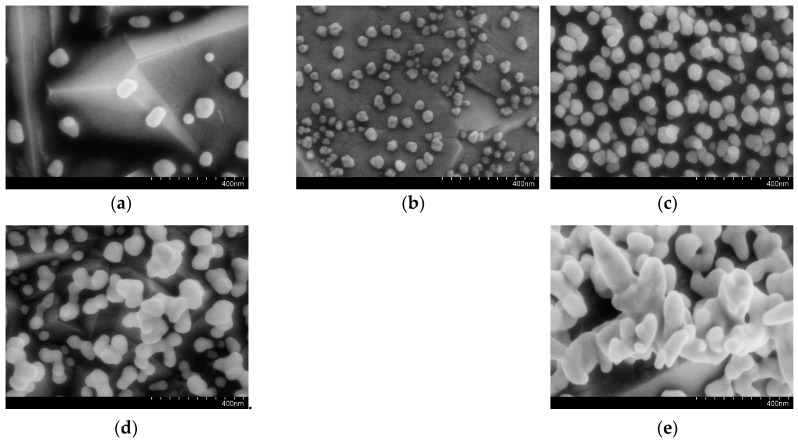
The SEM images of the AuNPs–BDD electrode with different deposition time (**a**) 100 s (**b**) 200 s (**c**) 300 s (**d**) 400 s (**e**) 500 s.

Gold nanoparticles were prepared by electrochemical reduction. As shown in Figure 9, the peak current reaches the maximum when the deposition time is 300 s. According to the electrode surface morphology characterization shown in Figure 10, this may be due to the increase in the number and size of AuNPs with the deposition time increasing in the range of 100 to 300 s, which provides more active and reduction sites on the electrode for Cr (VI). With the increase of the deposition time, the gold nanoparticles may form aggregations, and the electrode surface is not uniform, which results in a decrease in peak current [38]. The AuNPs deposition time of 300 s is selected for subsequent experiments.

**Figure 11:**
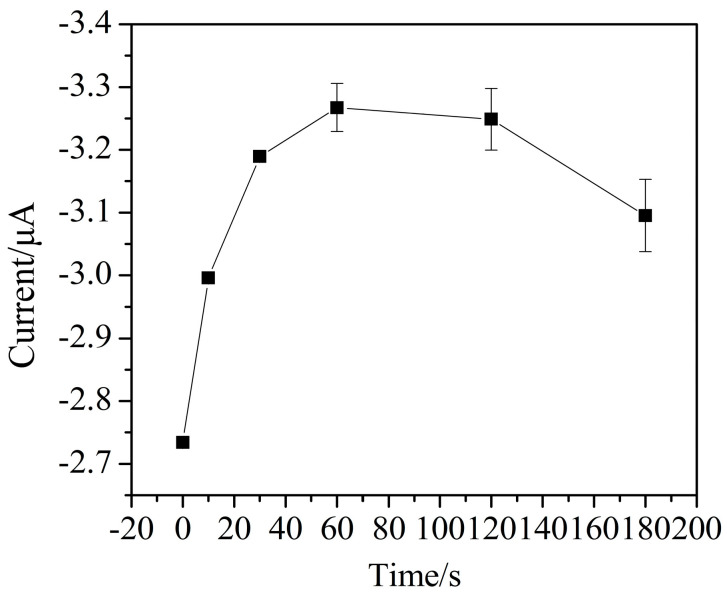
The effect of preconcentration time on the current response of Cr (VI) detection (pulse size = 25 mV, frequency = 25 Hz, step size = 5 mV, 0.1 mol/L sodium acetate buffer, pH 6).

When a preconcentration potential is applied to the electrode for a period of time, the Cr (VI) in the solution will be adsorbed to the electrode surface, so the preconcentration time has a great influence on the amount of Cr (VI) adsorbed on the electrode surface. The effect of preconcentration time on the peak current of Cr (VI) is shown in Figure 11. The measurements were obtained in 0.1 mol/L sodium acetate buffer using the following parameters—an initial potential of 0.6 V, a final potential of −0.1 V, and a preconcentration potential 0.6 V. The peak current increases from 0 to 60 s with the preconcentration time, and there is no obvious increase after 60 s. This may be due to the fact that the amount of Cr (VI) on the electrode surface increases within 0 to 60 s, which results in an increase of the Cr (VI) reduction current. After 60 s, the amount of Cr (VI) on the electrode surface reaches saturation. As the preconcentration time increases more than 60 s, the standard deviation of the detection current increases, and the detection current is unstable. So, the preconcentration time of 60 s is selected for subsequent experiments.

**Figure 12:**
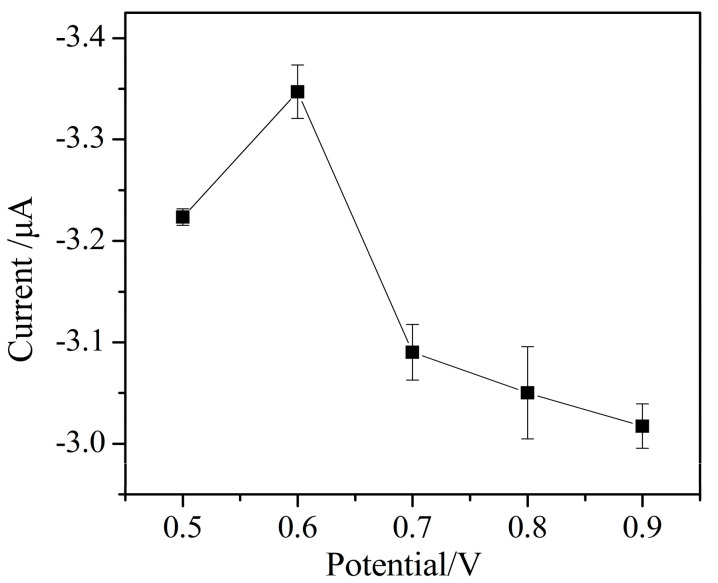
The effect of preconcentration potential on the current response of Cr (VI) detection (pulse size = 25 mV, frequency = 25 Hz, step size = 5 mV, 0.1 mol/L sodium acetate buffer, pH 6).

The effect of preconcentration potential on the peak current of Cr (VI) is shown in Figure 12. The peak current increases with the increase of the preconcentration potential in the range from 0.5 to 0.6 V. This is due to the amount of negatively charged Cr (VI) enriched on the electrode surface increasing at a positive potential. When the preconcentration potential is greater than 0.6 V, the peak current decreases. This may be due to other electrochemical active ions in the supporting electrolyte that also could be adsorbed on the electrode surface and compete with Cr (VI) ions at the high preconcentration potential, leading to decrease in the detection current of Cr (VI).

### 3.4. Detection of Cr (VI)

**Figure 13:**
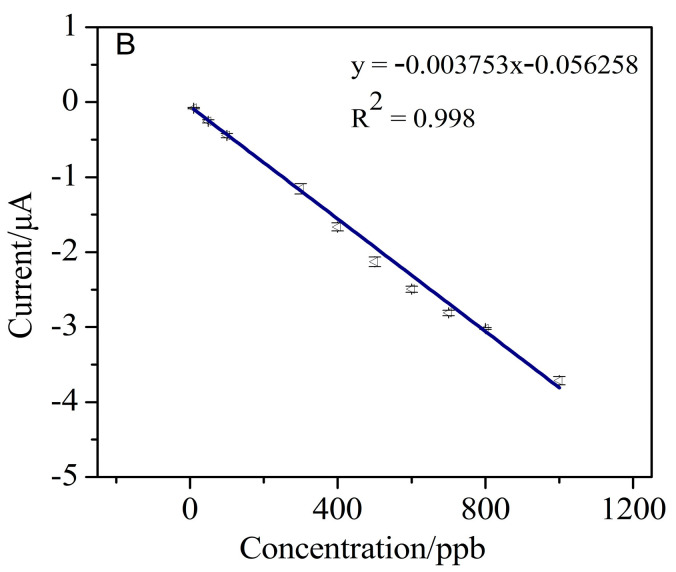
The response current curve for different concentrations of Cr (VI) solution of 0–1000 μg/L (pulse size = 25 mV, frequency = 25 Hz, step size = 5 mV, 0.1 mol/L sodium acetate buffer, pH 6, AuNPs deposition time 300 s).

Under optimized determination conditions, the detection performance of the AuNPs–BDD electrode for Cr (VI) in the concentration range of 10–1000 μg/L was studied. Figure 13 shows a linear behavior between the Cr (VI) concentration and the detection current, showing a good linearity between the concentration of Cr (VI) and the peak current. The linear correlation coefficient and sensitivity are 0.998 and 3.75 μA·mg^−1^·L, respectively. According to 3S_b_/S [39], a low limit of detection for Cr (VI) is calculated to be 1.19 μg/L. For the electrochemical detection of Cr (VI), the AuNPs–BDD electrode shows a high sensitivity and a low limit of detection. The relative standard deviation (RSD) of the five repetitive measurements for 500 μg/L Cr (VI) solution is 3.1%, indicating that the AuNPs–BDD electrode has good repeatability.

### 3.5. Selectivity of AuNPs–BDD Electrode

**Figure 14:**
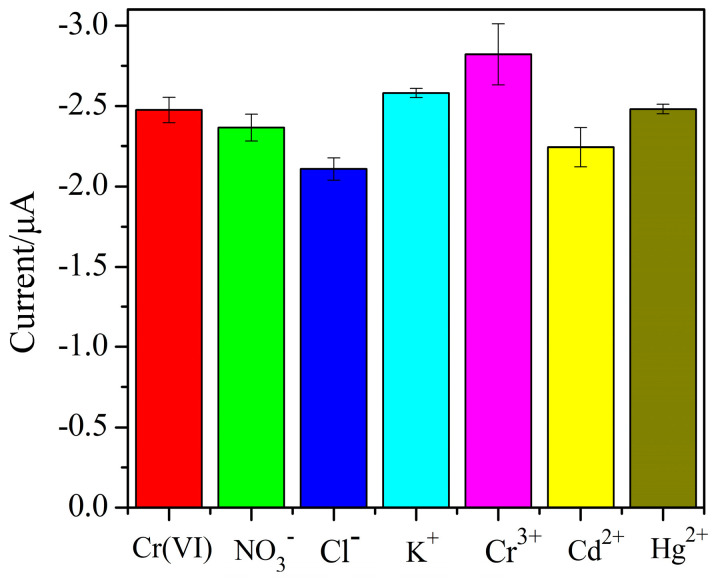
The influence of foreign ions on the current response of Cr (VI) detection.

This study investigated the selectivity of the AuNPs–BDD electrode for Cr (VI) detection. Foreign ions including Cl^−^, NO_3_^−^, K^+^, Cd^2+^, Hg^2+^, and Cr^3+^ were added to the Cr (VI) solution, and the detection was repeated three times. It can be seen from Figure 14 that a 5-fold excess of foreign ions has an acceptable interference effect on the detection current of Cr (VI). The result shows that the AuNPs–BDD electrode has good selectivity toward the detection of Cr (VI).

### 3.6. Real Sample Analysis

The AuNPs–BDD electrode was further investigated with tap water samples to evaluate the practical determination of Cr (VI). The water samples were adjusted to 0.1 mol/L sodium acetate buffer (pH 6). No trace of Cr (VI) was found when these samples were analyzed. The practical application performance of the sensor was investigated by the standard addition method, and the results are presented in Table 2. The result shows the acceptable recovery of tap water samples with 97.8–105.6%. It indicates that the sensor based on the AuNPs–BDD electrode could be a potential candidate for the practical detection of real samples.

### 3.7. Comparison with Other Methods

The performance of the AuNPs–BDD electrode was compared with several Cr (VI) electrochemical sensors. As shown in Table 3, the limit of detection for Cr (VI) in our work is better than that of most sensors. This is may be due to the low background current and good catalytic activity of the AuNPs–BDD electrode, which improves the electrochemical performance of the sensors.

## 4. Conclusions

Due to the excellent catalytic activity and low background current provided by AuNPs and BDD, the AuNPs–BDD electrode can be used for the sensitive and rapid determination of Cr (VI). The electrochemical detection of Cr (VI) on the AuNPs–BDD electrode is achieved by cathodic stripping voltammetry in the concentration range of 10–1000 μg/L at pH = 6, which has a good linearity and low detection limit with 1.19 μg/L. This method can detect Cr (VI) directly, and the detection process is simple and rapid, which does not require a complexing agent or catalyst. At the same time, the sensor is environmentally friendly and does not require an acidic environment during the detection process. Therefore, the AuNPs–BDD electrode has the potential for the rapid and on-site detection of Cr (VI). In the future, the renewal of the electrode surface and detection performance of onsite detection of Cr (VI) needs to be investigated further.

## Figures and Tables

**Table 1:** The effect of different electrolytes on the reduction current of Cr (VI).

Electrolytes	Reduction Peak Current (μA)
Nitric acid	-
Hydrochloric acid	-
Phosphate buffer	−3.14
Sodium acetate buffer	−8.74

**Table 2:** Recovery result for Cr (VI) in real water sample.

Heavy Metal	Added (μg/L)	Found (μg/L)	Recovery (%)
Cr (VI)	0	0	
	300	316.8 ± 23.4	105.6
	500	509.9 ± 19.7	102.0
	900	880.5 ± 10.9	97.8

**Table 3:** Comparison with other methods for the detection of Cr (VI).

Electrode	Method	Linear Range (ppb)	Limit of Detection (ppb)	Reference
Graphite/styrene–acrylonitrile copolymer composite electrode	SWASV	0–150	4.5	[40]
Polyoxometalate-based electrode	amperometry	25–18,900	2.7	[41]
AuNP–carbon nanotubes	amperometry	40–11,500	36	[42]
AuNPs–BDD	CSV	10–1000	1.19	This work
